# Intra- and Interspecies Spread of a Novel Conjugative Multidrug Resistance IncC Plasmid Coharboring *bla*_OXA-181_ and *armA* in a Cystic Fibrosis Patient

**DOI:** 10.1128/spectrum.03121-22

**Published:** 2022-09-26

**Authors:** Javier E. Fernandez, Helena M. B. Seth-Smith, Patrice Nordmann, Adrian Egli, Andrea Endimiani, Vincent Perreten

**Affiliations:** a Division of Molecular bacterial Epidemiology and Infectious Diseases, Institute of Veterinary Bacteriology, Vetsuisse Faculty, University of Bern, Bern, Switzerland; b Graduate School of Cellular and Biomedical Sciences, University of Bern, Bern, Switzerland; c Clinical Bacteriology and Mycology, University Hospital Basel, Basel, Switzerland; d Applied Microbiology Research, Department of Biomedicine, University of Basel, Basel, Switzerland; e Swiss National Reference Center for Emerging Antibiotic Resistance (NARA), University of Fribourg, Fribourg, Switzerland; f Institute for Infectious Diseases (IFIK), University of Bern, Bern, Switzerland; Universidad de Buenos Aires, Facultad de Farmacia y Bioquímica

**Keywords:** antibiotic resistance, OXA-48, *armA*, Enterobacterales, class D carbapenemases, 16S rRNA methylases, aminoglycoside-modifying enzymes, carbapenemase, cystic fibrosis, plasmid-mediated resistance

## Abstract

A novel multidrug resistance conjugative 177,859-bp IncC plasmid pJEF1-OXA-181 coharboring the carbapenemase-coding *bla*_OXA181_ and the aminoglycoside resistance 16S rRNA methyltransferase-coding *armA* genes was detected in two unrelated Escherichia coli gut isolates of ST196 and ST648, as well as two ST35 Klebsiella pneumoniae gut and sputum isolates of a cystic fibrosis patient. The *armA* gene was located within the antimicrobial resistance island ARI-A and the *bla*_OXA181_ gene, which was preceded by IS*903* and IS*Ecp1Δ* was inserted within the transfer genes region without affecting conjugation ability. Comparative plasmid analysis with other related IncC plasmids showed the presence of *bla*_OXA181_, as well as its integration site, are thus far unique for these types of plasmids. This study illustrates the potential of a promiscuous multidrug resistance plasmid to acquire antibiotic resistance genes and to disseminate in the gut of the same host.

**IMPORTANCE** Colocalization of carbapenemases and aminoglycoside resistance 16S rRNA methylases on a multidrug resistance conjugative plasmid poses a serious threat to public health. Here, we describe the novel IncC plasmid pJEF1-OXA-181 cocarrying *bla*_OXA-181_ and *armA* as well as several other antimicrobial resistance genes (ARGs) in different Enterobacterales isolates of the sputum and gut microbiota of a cystic fibrosis patient. IncC plasmids are conjugative, promiscuous elements which can incorporate accessory antimicrobial resistance islands making them key players in ARGs spread. This plasmid was thus far unique among IncC plasmids to contain a *bla*_OXA-181_ which was integrated in the transfer gene region without affecting its conjugation ability. This study highlights that new plasmids may be introduced into a hospital through different species hosted in one single patient. It further emphasizes the need of continuous surveillance of multidrug-resistant bacteria in patients at risk to avoid spread of such plasmids in the health care system.

## OBSERVATION

During the last 10 years, there has been an alarming spread of carbapenemase-encoding *bla*_OXA-48_-like genes among Gram-negative clinical isolates worldwide (1). Among them, *bla*_OXA-181_ is the second most prevalent and its dissemination has been mainly driven by a conjugative ~51 kb IncX3 pandemic plasmid which carries *bla*_OXA-181_ on a 14-kb transposon (2). Further mobile genetic elements (MGE) harboring *bla*_OXA-181_ have been identified (such as ColE2, IncN1, and IncT plasmids), but less frequently (1, 3). Moreover, plasmid-mediated co-occurrence of carbapenemases with 16S rRNA methylases, which confer high level resistance to all clinically available aminoglycosides, has been also increasingly reported in Switzerland (4), further limiting therapeutic options against multidrug-resistant (MDR) and carbapenemase-producing *Enterobacterales* (CPE) (5–7). Of concern, the colocalization of multiple antimicrobial resistance genes (ARGs) on the same promiscuous conjugative plasmids represents a serious risk for rapid dissemination of antibiotic resistance within the bacterial population in individuals and clinical settings.

During a routine screening of a 25-year-old cystic fibrosis (CF) patient in 2019 for multidrug-resistant Gram-negative bacteria at the University Hospital Basel in Switzerland, Escherichia coli and Klebsiella pneumoniae were identified in rectal and sputum samples. The isolates were obtained the same day on selective agar plates (chromID CARBA SMART, bioMérieux). From the rectal swab sample, two E. coli morphotypes (806883-9-2019 and 806883-11-2019) and one K. pneumoniae morphotype (806883-10-2019) were selected and identified with routine MALDI-TOF MS (Bruker). K. pneumoniae (503666-2-2019) was isolated from the sputum sample. These isolates were submitted for antimicrobial susceptibility testing (AST) and whole-genome sequencing (WGS) to determine their phylogenetic background and ARGs. The patient declared not to travel, but has a long history of antimicrobial consumption related to the underlying chronic disease. The strains were resistant to carbapenem (ertapenem), cephalosporins (cefepime, cefotaxime), aminoglycosides (gentamicin, amikacin), sulfonamides, and trimethoprim as determined by broth microdilution techniques using Thermo Scientific Sensititre EUVSEC panels and EUCAST recommendations (www.eucast.org), and still susceptible to ceftazidime-avibactam as determined by gradient diffusion test (Liofilchem) (Table 1).

**TABLE 1:** Sequencing coverage and MICs of antibiotics for Klebsiella pneumoniae ST35 and Escherichia coli ST196 and ST648 as well as an E. coli J53dR transconjugant containing plasmid pJEF1-OXA-181

WGS statistics and antimicrobials	K. pneumoniae ST35 (503666-2-2019)	K. pneumoniae ST35 (806883-10-2019)	E. coli ST196 (806883-11-2019)	E. coli ST648 (806883-9-2019)	E. coli J53dR with pJEF1-OXA-181
Illumina coverage	299×	387×	61×	37×	-^*b*^
ONT coverage	128×	156×	211×	256×	162×
ONT N50	16,000	15,000	14,700	17,000	17,300
MIC (in μg/Ml) of antibiotics:					
Cefoxitin	64	16	32	32	32
Ertapenem	2	2	1	2	0.5
Imipenem	1	0.5	0.5	1	1
Meropenem	1	1	0.25	1	0.25
Ceftazidime	128	8	32	8	32
Cefepime	1	0.5	0.5	0.5	0.25
Cefotaxime/clavulanic acid	32/4	4/4	32/4	4/4	16/4
Ceftazidime/clavulanic acid	128/4	8/4	32/4	8/4	32/4
Cefotaxime	32	4	32	4	32
Temocillin	>128	>128	>128	>128	>128
Ampicillin	>32	>32	>32	>32	>32
Ciprofloxacin	0.06	0.06	0.03	0.03	≤0.015
Azithromycin	64	64	>64	32	64
Amikacin	>128	>128	>128	>128	>128
Gentamicin	>16	>16	>16	>16	>16
Tigecycline	0.5	0.5	≤0.25	0.5	≤0.25
Chloramphenicol	≤8	≤8	≤8	≤8	≤8
Colistin	≤1	≤1	≤1	≤1	≤1
Nalidixic acid	≤4	≤4	≤4	≤4	≤4
Tetracycline	≤2	≤2	≤2	≤2	≤2
Trimethoprim	>16	>16	>16	>16	>32
Sulfamethoxazole	>512	>512	>512	>512	32
Ceftazidime/avibactam^*a*^	0.50	0.25	0.38	0.19	0.19

a As determined by gradient diffusion test (Liofilchem) on Muller-Hinton agar plates.

b -, Illumina sequencing not performed.

The isolates were sequenced using both short- (Nextseq500 Illumina, 2 × 150 bp paired-end, coverage in Table 1) and long-read (MinION, Oxford Nanopore Technologies) technologies. Raw reads were quality controlled using FASTQC and quality-based trimmed (short-reads) with Trimmomatic. The genome of each strain was *de novo* assembled with the hybrid approach of Unicycler v0.4.9b using both sets of reads to obtain high-quality circular contigs. The completeness of the contigs was confirmed by read mapping the long reads using Minimap2 v2.17. Core genome MLST (cgMLST) relatedness was determined using Ridom SeqSphere+ v 8.3.1, and schemes described at https://cgmlst.org/. Coding sequences (CDSs) were predicted and annotated using Prokka v1.14.6 and the NCBI Prokaryotic Genome Annotation Pipeline (PGAP) upon submission, and manually curated by BLASTP analysis. The genome assemblies were screened for ARGs *in silico* using the command-line version of ResFinder v4.0 and rgi v5.2.0 against the CARD database v3.1.4, and the identified plasmids were replicon-typed with PlasmidFinder v2.1. Multiple sequence alignment and pairwise nucleotide comparisons were carried out using Mauve v1.1.3 (progressiveMauve algorithm).

**FIG 1:**
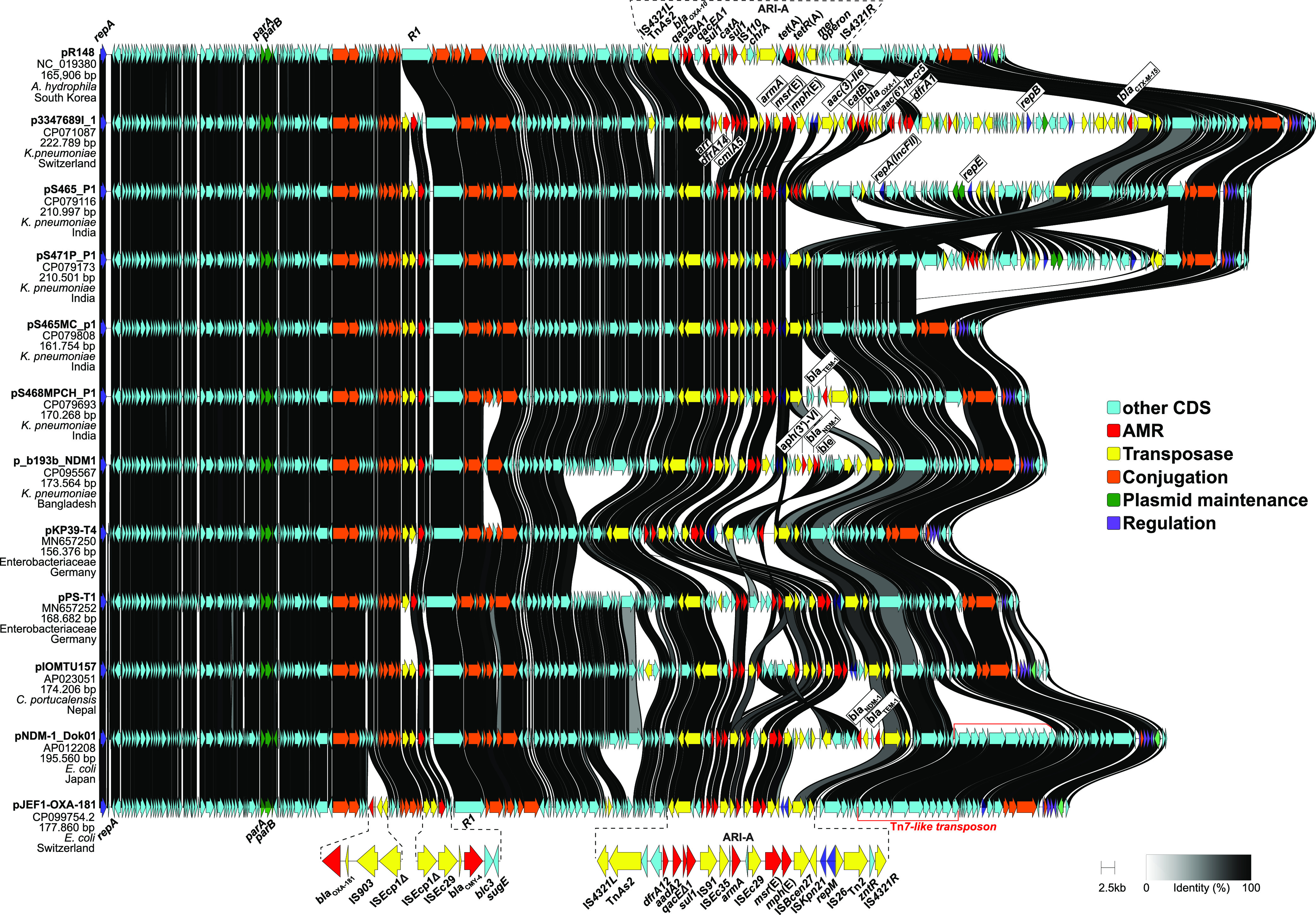
Gene cluster comparison of the 177,859-bp plasmid pJEF1-OXA-181 with the IncC type 1a reference plasmid pR148 (GenBank acc. no NC_019380) and the 10 closest related plasmids from the GenBank (>80% coverage and >99,99% identity) (accessed August 10, 2022). Plasmid names, bacterial host, country of origin, and corresponding GenBank accession numbers are indicated for each plasmid. The AMR-carrying regions of pJEF1-OXA-181 are enlarged for better visualization. CDS are portrayed as arrows with colors indicating specific functions. AMR, antimicrobial resistance gene. Graphical representation was created with clinker v0.0.23.

All isolates contained a novel 177,859-bp multidrug resistance IncC plasmid named pJEF1-OXA-181 (Fig. 1). IncC plasmids possess a conserved backbone and can be differentiated into two types, namely 1 (subtypes a and b) and 2 (6). They usually carry several additional insertions, including accessory antimicrobial resistance islands (ARI) (Fig. 1). Plasmid pJEF1-OXA-181 belongs to type 1, subtype a and carries a 26,698-bp antibiotic resistance island (ARI-A) flanked by IS*4231* as well as *bla*_OXA-181_ and the plasmid-mediated AmpC cephalosporinase gene *bla*_CMY-4_ on IS*Ecp1* transposition units. ARI-A harbors genes known to confer resistance to aminoglycosides, gentamicin and amikacin (*armA*), streptomycin and spectinomycin (*aadA2*), macrolides (*msr*(E), *mph*(E)), sulfonamides (*sul1*), trimethoprim (*dfrA12*), and disinfectants (*qacEΔ1*) inserted downstream of the primase gene (ARI-A insertion site) (Fig. 1).The 5,007-bp *bla*_CMY-4_-containing element (IS*Ecp1Δ*-IS*Ec29*-*bla*_CMY-4_-*blc*-*sugE*) was integrated into pJEF1-OXA-181 downstream of *traA*. The *bla*_OXA-181_ was also located downstream of a truncated IS*Ecp1* in a 3,910-bp element (IS*Ecp1Δ*-IS*903*-*bla*_OXA-181_), which was inserted 1,480 bp downstream of *traD*. Plasmid pJEF1-OXA-181 contained the complete IncC machinery for conjugation (*traIDLEKBVACWUNFHG* and *trhF*) (6) (Fig. 1) and was confirmed to be transferable from E. coli and K. pneumoniae donors to E. coli J53dR by filter mating experiment at a rate of 6.8 × 10^−5^ and 1.6 × 10^−3^ transconjugants per donor, respectively, as described previously using 2 μg/mL of ceftazidime in selective plates (8) (Table 1).

Multiple alignment of the sequences of pJEF1-OXA-181 from all four strains revealed minor differences among the plasmid in each strain. Using pJEF1-OXA-181 of E. coli 806883-11-19 as reference, five single nucleotide polymorphisms (SNPs) and 1-bp insertion were found in the plasmid of E. coli 806883-9-19. In the two K. pneumoniae, pJEF1-OXA-181 differed by one SNPs, and by five SNPs in 806883-10-19 and by four SNPs in 503666-2-19 to the plasmid of E. coli 806883-11-19. These differences may be due to the independent evolution, i.e., SNPs accumulation of pJEF1-OXA-181 in the different host species. Since its detection in this patient in 2019, pJEF1-OXA-181 was neither detected in any of the other OXA-181 containing isolates identified in the hospital (*n* = 4) nor among the 69 *bla*_OXA-181_-containing E. coli strains deposited to the Swiss National Reference Center for Emerging Antibiotic Resistance (NARA) in Switzerland. The 10 closest related plasmids from the GenBank shared the backbone of the IncC type 1a plasmids, but differ in their ARGs carriage (Fig. 1). Among them, plasmid pNDM-1_Dok01 reported in Japan (GenBank acc. no. AP012208.1) was the most similar sharing most of the resistance modules (*bla*_CMY-4,_ *armA*, *aadA2*, *msr*(E), *mph*(E), *sul1*, *dfrA12*). However, it carries *bla*_NDM-1_ and lacks the element containing *bla*_OXA-181_ (Fig. 1).

E. coli 806883-9-19 belongs to ST648 which is an extraintestinal pathogenic (ExPEC) lineage and 806883-11-19 to ST196; they differ by 2,368 loci (of the 2,513 used in the scheme). Both K. pneumoniae share the same cgMLST allelic profile (0 allele differences, based on the 2,358 loci scheme) and belong to ST35, which is an international clone (9). In addition to pJEF1-OXA-181, the K. pneumoniae isolates also carry an IncFIB(K) plasmid harboring the extended-spectrum β-lactamase CTX-M-44 and the recently described narrow-spectrum β-lactamase OXA-926 (10).

These results indicate that multiple ARGs, including those conferring resistance to last resort antibiotics, have accumulated on a broad host range conjugative IncC plasmid. These plasmids play an important role in the dissemination of resistance as they usually carry several resistance modules, including cocarriage of carbapenemase (*bla*_NDM_, *bla*_IMP_, *bla*_VIM_, *bla*_KPC_) and aminoglycoside resistance 16S rRNA methyltransferase (*armA*, *rmtA-H*) genes (6, 11). However, the copresence of *bla*_OXA-181_ and *armA* on such plasmids and the insertion site of the *bla*_OXA-181_ containing module in the conserved backbone is thus far unique. Intra- and interspecies dissemination of plasmids containing carbapenemase genes such as *bla*_KPC-3_ (12) and *bla*_OXA-48_ (13) in a host microbiome have already been reported. This phenomenon emphasizes the importance of screening of patient at risk in order to apply infection control procedures to avoid possible introduction and dissemination of such plasmids within a hospital. Indeed, the spread of the multidrug resistance IncC plasmid pJEF1-OXA-181 in different Enterobacterales within the same patient underlines its promiscuity and potential to rapidly disseminate in clinical settings.

### Data availability.

Complete genomes of E. coli 806883-9-2019 and 806883-11-2019 and K. pneumoniae 806883-10-2019 and 503666-2-2019 have been deposited in the GenBank (BioProject: PRJNA850540) under accession numbers CP099754, CP099755, CP099756, CP099757, CP099758, CP099759, and CP099752-CP099753, and CP099748, CP099749, CP099750, CP099751, and CP099743, CP099744, CP099745, CP099746, CP099747, respectively.
